# Ten Consecutive Laparotomies

**Published:** 1889-08

**Authors:** Virgil O. Hardon

**Affiliations:** Professor of Obstetrics and Diseases of Women and Children, Atlanta Medical College, Atlanta, Ga.


VOL. VI. AUGUST, 1889. No. 6.

### Original Communications.

### TEN CONSECUTIVE LAPAROTOMIES.

BY VIRGIL O. HARDON, M. D.,

Professor of Obstetrics and Diseases of Women and Children, Atlanta Medical College, Atlanta, Ga.

CASE I.—LAPAROTOMY FOR PELVIC ABSCESS. RECOVERY.—H. Z., married, aged 22, no children; two miscarriages, the last four years ago. Since that time has suffered from recurrent attacks of pelvic pain, tenderness in the groins and profuse leucorrhœa. In April, 1887, after such an attack, she noticed a swelling in the left groin, painful to touch, which gradually increased in size. She came under my care July 24, 1887. She was unable to walk on account of pain, emaciated, without appetite, pulse 120, temperature 101. In the left groin was a rounded swelling, obvious to inspection. By vaginal examination a painful and tender fluctuating mass could be felt, extending down into the pelvis and pushing the uterus completely over to the right side. Aspiration showed that it contained pus.

*Operation, July 31, 1887.*—Present Drs. Cooper, Harris and Greene. The abdomen was opened by an incision two inches long, immediately over the tumor, midway between the median line and the left anterior superior spinous process. The pus sac was not adherent to the abdominal wall. The abscess was located in the left broad ligament. An incision through its wall gave exit to about a quart of fetid pus, some of which escaped into the abdominal cavity. The abdomen and the pus sac were thoroughly washed out and a drainage tube inserted into each. The edges of the pus sac were stitched to the edges of the abdominal incision. Time of operation, fifty minutes. The patient recovered well from the operation. Highest temperature 101.4 on the afternoon of second day. The pus sac was washed out daily and packed with sublimate gauze. It slowly filled by granulation, and on November 1, was completely closed. The patient that time was fully restored to health and had gained over at thirty pounds in weight since the operation.

CASE II.—SALPINGO-OOPHORECTOMY. DEATH.—D. Q., married, age 34. Never has been pregnant. Menstruated at 17. About six months after went bathing while menstruating, which caused her periods to become scanty and painful. Married at 20. About six months after marriage had an attack of pelvic inflammation, which was followed by a discharge of pus from the unbilicus, which continued in varying quantity until six months ago. For the last five years has had frequent attacks of pelvic inflammation, which have induced a state of chronic invalidism. The last attack reduced the patient to a desperate condition of weakness, and in this state she came under my care at the commencement of a fresh attack. Examination showed an enlarged fallopian tube on the left side, surrounded by extensive inflammatory deposit.

*Operation, September 15, 1887.*—Present Drs. Todd, Cooper and Harris. Incision in median line. Left tube distended with pus and embedded in inflammatory deposit. Separated and removed with great difficulty, together with left ovary. Right tube and ovary apparently normal, therefore not removed. Free hemorrhage, controlled by sponging. Abdomen washed out with hot water and closed without drainage. Time of operation, one hour and ten minutes. Patient put to bed in a condition of collapse and died of shock two hours after the operation.

CASE III.—BATTEY'S OPERATION FOR UTERINE FIBROID. RECOVERY.—P. F., married, age 34. Never has been pregnant. About three years ago noticed a tumor in the abdomen just to the right of median line, which has increased to the size of a fœtal head. For the last five months she has suffered severe pelvic pain and frequent profuse hemorrhages, by which her health has been so reduced that she has been unable to follow her vocation of a cook. Examination showed a sessile, fibroid tumor, as large as a foetal head, attached to the *fundus uteri.*

*Operation, April 25, 1888.*—Present Drs. Bowne, Cooper, Harris and Greene and Mr. Roy (M. S.) Incision in median llne, both ovaries removed, normal in appearance, no adhesions. Abdomen washed out with Thiersch's solution and a drainage tube inserted. Time of operation, forty minutes. Recovery uneventful. Highest temperature 100.4, on the afternoon of second day. She has not menstruated since the operation and the pelvic pain has entirely ceased. Fourteen months after the operation the tumor had diminished in size more than one-half. Patient is fully restored to health.

CASE IV.—BATTEY'S OPERATION FOR UTERINE FIBROID. RECOVERY.—M. F., single, age 28. Has suffered over a year with severe and constant pelvic pain and profuse menorrhagia, by which her general health has been much reduced, until she is unable to walk at all or work for her living. Examination showed a sessile, subserous, uterine fibroid as large as a goose egg, growing from the posterior aspect of the *fundus uteri.*

*Operation, May 2, 1888.*—Present Drs. Bowne, Cooper, Harris and Greene. Incision in median line, both ovaries removed, normal in appearance. No adhesions. Abdomen closed without washing or drainage. Time of operation, thirty-five minutes. Recovery uneventful. Highest temperature 100.6, on the afternoon of second day. Complete relief of menorrhagia and of pelvic pain. When examined at the end of a year the fibroid tumor was barely perceptible as a thickening of the posterior uterine wall.

CASE V.—BATTEY'S OPERATION FOR EXCESSIVE DYSMENORRHŒA. DEATH.—A. A., single, age 27. Has suffered since puberty from excessive pain at the menstrual periods. The pain has none of the features of obstructive dysmenorrhœa but appears to originate in the ovaries. Her health has given way, all her functions are impaired and she has been bedridden for two years. She has been under the care of competent physicians and all means of relief have been exhausted. Removal of the ovaries was offered as a last resort.

*Operation, June 20, 1888.*—Present Drs. Cooper, Harris, Greene and King of Texas. Incision in the median line. Both ovaries removed, apparently normal. Abdomen washed out with Thiersch's solution and drainage tube inserted. Time of operation, forty minutes. Peritonitis developed on the third day. Epsom salts in large doses was given, but without producing any action of the bowels. Tympanites, vomiting and exhaustion followed and patient died on the fifth day. Highest temperature 101.4, on day of death.

CASE VI.—EXPLORATORY INCISION. RECOVERY.—F. W., married, age 36. Never has been pregnant. About four years ago she noticed a "lump" in her abdomen, immediately above the pubic bone. This has gradually increased in size up to the present time. About fifteen months ago she began to have recurrent attacks of pelvic inflammation, which became more frequent, until at the present time her abdomen is constantly painful and tympanitic. The menses have become frequent and profuse. She is bedridden and emaciated to a skeleton; pulse ranging from 120 to 140, and temperature from 101 to 105. She is obliged to take large doses of morphine to enable her to endure the severe pain. Examination shows a fibroid tumor, blocking up the entire pelvis and extending half way to the umbilicus. There is also some ascites.

*Operation, October 5, 1888.*—Present Drs. Bowne, Harris, Greene and Mr. White (M. S.) Abdomen opened in median line. About a quart of ascitic fluid escaped. The fibroid tumor, which was as large as a fœtal head, was found to be universally adherent on all sides, and the tubes and ovaries were so embedded in inflammatory deposit that it was impossible to reach or even to locate them. The abdomen was thoroughly washed out with Thiersch's solution and closed without drainage. Time of operation, twenty-five minutes. Patient made a good recovery with the exception of an attack of peritonitis on the fourth day, which was checked in twenty-four hours by drachm doses of Epsom salts every hour until watery stools were produced. Highest temperature 101, on the afternoon of the fourth day. Since the operation she has been free from pain and there has been no recurrence of pelvic inflammation. She has gained thirty-eight pounds in weight, is able to walk wherever she chooses and feels perfectly well. There has been no apparent change in the size of the tumor and no return of the ascites.

CASE VII.—BATTEY'S OPERATION FOR PERSISTENT PELVIC PAIN. RECOVERY.—O. H., widow, age 38. One child five years ago. Always enjoyed good health until two years ago, when a cancer developed in the left breast which was subsequently removed. About September, 1888, she began to suffer for the first time with painful menstruation, the pain being in the ovarian region on both sides, but greater on the left. This pain increased in intensity and duration until, when I first saw her in November, it was constant and so severe as to require large doses of morphine every day. Vaginal examination revealed enlargement and tenderness of both ovaries, but nothing else abnormal in the pelvis. As all remedies were absolutely without avail and the patient was rapidly lapsing into the morphine habit, Battey's operation was offered and accepted.

*Operation, December 8, 1888.*—Present Drs. Cooper, Westmoreland, Jr., Jones and Johnson. Incision in median line, both ovaries removed. No adhesions. The left ovary contained a serous cyst as large as a hickory nut, and an hæmatic cyst slightly smaller. The right ovary contained several small cysts. Both ovaries were enlarged. The abdomen was sponged out and a drainage tube inserted. Time of operation, thirty-five minutes. Recovery uneventful. Highest temperature 100, on the afternoon of second day. Patient was free from pelvic pain until May, 1889, at which time a return of the malignant growth in the breast necessitated an operation for its removal. This operation was followed by a single recurrence of the menses with the same pain as before. Since that time there has been no return of the menses or of the pain.

CASE VIII.—SALPINGO-OOPHORECTOMY. RECOVERY.—J. B., married, age 28. Never pregnant. For three years she has suffered from recurrent attacks of pelvic inflammation, by which her health has been undermined and she has become unable to work. Examination shows the fallopian tubes on both sides largely dilated, filled with fluid and surrounded by inflammatory deposit.

*Operation, December 15, 1888.*—Present, Drs. Harris and Jones and three medical students. Incision in median line. Both tubes were found enlarged to the size of sausages and filled with pus, and, together with the ovaries, embedded in inflammatory deposits. By patient and careful dissection with the fingers, the organs were isolated and removed. Much hemorrhage followed, which was controlled by sponges. The abdomen was washed out by pouring in successive pitcherfuls of hot water, until it returned clear. A drainage tube was inserted. Time of operation, one hour. Patient did well until the fourth day, when peritonitis developed, which lasted forty-eight hours and was checked by drachm doses of Epsom salts every hour until watery stools were induced. Further recovery uneventful. Highest temperature 101.8, on morning of fifth day, Since the operation, patient has had no return of pelvic inflammation, has fully recovered her health and is now able to work for her living.

CASE IX.—SINGLE OVARIOTOMY. RECOVERY.—H. L., married, age 42. Always enjoyed good health until about two years ago, when she noticed a swelling in the right inguinal region. This gradually increased in size, until, at the present time, it fills and distends the whole abdomen and reaches to the diaphragm During the past six months her health has rapidly failed She is very emaciated and presents the characteristic *facies ovariana.* Diagnosis—unilocular cyst of right ovary.

*Operation, March 19, 1889.*—Present, Drs. Bowne, Pinckney, Cooper, Harris and Jones. Incision in median line. No adhesions. Fluid evacuated by trocar and sac readily withdrawn. Pedicle very large so that tying was not deemed safe. The stump was therefore brought up into the abdominal incision and stitched to the edges of it. The left ovary was normal, and was therefore allowed to remain. The abdomen was washed out by several pitcherfuls of hot water and a drainage tube inserted. Time of operation, one hour and twenty minutes. Weight of tumor, thirty-seven and a half pounds. Made a good recovery, the highest temperature being 100.8, on the afternoon of the second day. The stump in the abdominal opening slowly granulated over, and was not entirely healed until four months after the operation. Patient completely regained her health in the meantime.

CASE X.—BATTEY'S OPERATION FOR UTERINE FIBROID. RECOVERY.—C. N., married, age 32. Never has been pregnant. About four years ago first noticed a tumor in the abdomen, which has increased in size to the present time. For the past year has suffered from metrorrhagia and pelvic pain. For the last two months the pain has been so severe as to confine her to her bed and require the constant use of opiates. Examination showed an interstitial, fibroid tumor as large as a cocoanut.

*Operation, May 11, 1889.*—Present, Drs. Cooper, Harris, Smith and Benedict, of Athens, Ga. Incision in median line. Both ovaries removed and found to be enlarged to twice the normal size. In the right ovary was embedded a mass of apparently true bone as large as a hickory nut. Abdomen sponged out and a drainage tube inserted. Time of operation, twenty-five minutes. Recovery uneventful. Highest temperature 99.8, on the afternoon of second day. Since the operation there has been no return of metrorrhagia or pelvic pain. Menstruation occurred normally one month after the operation, lasting six days.

In all these operations strict antisepsis was observed with the exception of Case I. The drainage tube when used was of rubber, and was removed on the morning of the third day. No opium was given except in Case V, which died. All ligatures and sutures were of carbolized, iron-dyed, braided silk. The abdominal incision was closed by sutures passed through the whole thickness of the abdominal wall, from within outward. These were removed on the ninth or tenth day. I have had no case of stitch-hole abscess or of hernia. No food or drink was given for the first twenty-four hours; on the second day water, milk and ice, and afterwards as much nourishing food as possible. The bowels were moved on the third day by a dose of castor oil, except in Case V.

